# Ancient Patrilineal Lines and Relatively High ECAY Diversity Preserved in Indigenous Horses Revealed With Novel Y-Chromosome Markers

**DOI:** 10.3389/fgene.2020.00467

**Published:** 2020-05-21

**Authors:** Shuqin Liu, Yunzhou Yang, Qingjie Pan, Yujiang Sun, Hongying Ma, Yu Liu, Min Wang, Chunjiang Zhao, Changxin Wu

**Affiliations:** ^1^College of Animal Science and Technology, China Agricultural University, Beijing, China; ^2^Equine Center, China Agricultural University, Beijing, China; ^3^School of Animal Science and Technology, Qingdao Agricultural University, Shandong, China; ^4^National Engineering Laboratory for Animal Breeding, Beijing, China; ^5^Key Laboratory of Animal Genetics, Breeding and Reproduction, Ministry of Agriculture, Beijing, China; ^6^Beijing Key Laboratory for Animal Genetic Improvement, Beijing, China

**Keywords:** horse, patrilineal lines, Y-chromosome, SNP, CNV

## Abstract

Extremely low nucleotide diversity of modern horse Y-chromosome has been reported, and only poor phylogenetic resolution could be resulted from limited Y-chromosome markers. In this study, three types of horse Y-chromosome markers, including Single-nucleotide polymorphisms (SNPs), copy number variants (CNVs), and allele-specific CNVs, were developed by screening more than 300 male horses from 23 indigenous Chinese horse populations and 4 imported horse breeds. Fourteen segregating sites including a novel SNP in the AMELY gene were found in approximately 53 kb of male-specific Y-chromosome sequences. CNVs were detected at 11 of 14 sites, while allele-specific CNVs at 6 polymorphic sites in repeated fragments were also determined. The phylogenetic analyses with the SNPs identified in this study and previously published 51 SNPs obtained mainly from European horses showed that indigenous Chinese horses exhibit much deeper divergence than European and Middle Eastern horses, while individuals of Chinese horses with the C allele of the AMELY gene constituted the most ancient group. Via SNPs, CNVs, and allele-specific CNVs, much higher diversity of paternal lines can be detected than those identified with merely SNPs. Our results indicated that there are ancient paternal horse lines preserved in southwestern China, which sheds new light on the domestication and immigration of horses, and suggest that the priorities of the conservation should be given to the ancient and rare paternal lines. These three marker types provided finer phylogenetic resolution of horse patrilineal lines, and the strategies used in the present study also provide valuable reference for the genetic studies of other mammalian patrilineages.

## Introduction

In many mammalian species, patrilines are much less than maternal lines. At the DNA level, patrilines show low diversity in Y-chromosome sequences, in contrast to high level of variation in mitochondrial sequences (Marquez et al., 2010; Cortes et al., 2011; Bidon et al., 2014; Hallast and Jobling, 2017). Equids are the typical animals with limited paternal lines. Studies of the mitochondrial D-loop in modern horse breeds have revealed high matrilineal diversity without a strong phylogeographic structure (Lister et al., 1998; Vila et al., 2001; Jansen et al., 2002; Lei et al., 2009; Cieslak et al., 2010; Lira et al., 2010). The mtDNA and autosomal microsatellite DNA data suggested multiple domestications and introgressions of wild females (Vila et al., 2001; Jansen et al., 2002; Achilli et al., 2012; Warmuth et al., 2012). In contrast to the abundant maternal diversity found in domestic horses, extremely low nucleotide diversity has been detected in the Y chromosome of modern horses (Lindgren et al., 2004; Rafeie et al., 2011; Brandariz-Fontes et al., 2013), and though data from an analysis of ancient DNA suggest that the Y chromosome diversity in ancient wild horses was higher than that of extant horses and other wild mammals (Lippold et al., 2011). Two haplotypes (HTs) of a Y chromosome-specific microsatellite DNA locus in indigenous Chinese horses were reported in 2010, which was the first study in which genetic variants of the horse Y chromosome (*Equus caballus* Y chromosome, and ECAY) were found (Ling et al., 2010). Later, two additional male lineages of domestic horses, revealed with Y-chromosome microsatellite polymorphisms, were described (Kreutzmann et al., 2014). In recent years, Wallner et al. intensively studied Y-chromosome variants of European horses and made significant progress in the identification of modern horse paternal lineages. They successfully identified a number of new variants by screening 1.46 Mb of male-specific Y regions of 21 European breeds, 2 American breeds, and 6 Asian breeds, which greatly facilitate the phylogenetic study of paternal horse populations (Wallner et al., 2017; Felkel et al., 2018, 2019).

The extremely low Y chromosome diversity in extant horses has been mainly attributed to intensive breeding practices after domestication, and the Y chromosome diversity decreased dramatically because of positive selection (Levine, 1999; Cunningham et al., 2001; Wallner et al., 2004; Lippold et al., 2011). The low level of diversity of horse Y chromosome sequences makes it difficult to specifically discriminate among stallions and leads to low phylogenetic resolution of horse patrilineal lines, which limits their application in the evaluation of patrilineal diversity in horses. Therefore, it is essential to develop novel markers for genetic studies and conservation purposes of horse patrilineages.

Most horse populations in the world have been subjected to intensive artificial selection and genetic introgression over the past several centuries (Hendricks, 2007). However, certain Chinese indigenous horse populations have largely preserved their genetic diversity because they have not been subjected to intensive selection (Lei et al., 2009; Chang, 2011; Jiang et al., 2011; Ling et al., 2011; Xu et al., 2012; Yue et al., 2012), and they have now become valuable and ideal extant horse populations for studying ECAY diversity. There are over 3 million horses in China (Chang, 2011), and the majority of them are indigenous, making them among the largest gene pools of indigenous horses in the world. They are widely distributed in many provinces, with the largest populations inhabiting the mountainous southwestern provinces (primarily south of the Yangtze River, including Guangxi, Yunnan, Guizhou, and Sichuan) as well as the northern provinces, such as Inner Mongolia, Xinjiang, Gansu, and Jilin. North China has a history of horse husbandry that spans over 3,000 years (Yuan, 2004). It has been proposed that domestic horses in North China originated outside of China and were gradually introduced from the Eurasian steppe during the Bronze Age (He, 2008), and those in South China were introduced by ancient people who immigrated from northern and northwestern areas of China (Zhang, 1984; Hou, 1990).

Previous studies have demonstrated that Chinese indigenous horses harbor unique ECAY microsatellite DNA variants and SNP HTs (Hou, 1990; Ling et al., 2010; Han et al., 2015; Han et al., 2019). In the present study, novel Y-chromosome markers, consisting of Single-nucleotide polymorphisms (SNPs), copy number variants (CNVs), and allele-specific copy number variants (AS-CNVs), were developed by screening more than 300 male horses including both indigenous Chinese horses and foreign horses with DNA sequencing, quantitative PCR, and pyrosequencing, respectively. The markers were further used in the phylogenetic study of horse patrilineal lineages, which shed new light on the paternal lines of domesticated horses and provide a novel strategy for studying and assessing the diversity of paternal lines.

## Materials and Methods

### Sampling and DNA Extraction

A total of 235 male horse blood samples from 23 Chinese indigenous horse populations were collected. Sixty-six samples of foreign male horses, consisting of 31 Thoroughbreds (TB), 15 Arabian horses (AR), 13 Akhal-Tekes (AT), and 7 Warmbloods, were provided by the China Stud Book. A *Przewalski’s* horse was also sampled as an outgroup. Detailed information of the sampled populations is described in Table 1 and Supplementary Figure S1. Genomic DNA was isolated using standard phenol/chloroform extraction methods, and DNA samples were diluted to 50 ng/μl and served as templates for PCR. The samples were obtained following the principles approved by the Animal Care and Use Committee of China Agricultural University.

**TABLE 1:** ECAY genetic diversity of Chinese indigenous horse populations.

**Breed**	**Abbreviations**	**n/N**	**HT**	**π**	**h**
inter-Mongolia	IMG	2/7	SHT1, SHT2	0.00030.00002	0.2860.196
Yanqi	YQ	1/8	SHT2	0.00000.0000	0.000.00
Kazakh	KZK	2/12	SHT1, SHT2	0.000050.00001	0.485000.10600
Chakouyi	CK	3/9	SHT1, SHT2, SHT18	0.000030.00002	0.417000.19100
Elunchun	ELC	3/10	SHT1, SHT2, SHT3	0.000050.00001	0.5330.09500
Xinihe	XNH	3/7	SHT1, SHT2, SHT3	0.00030.00002	0.2860.196
Langkazi	LKZ	2/5	SHT1, SHT17	0.000020.00001	0.40.237
Jiangzi	JZ	5/7	SHT1, SHT4, SHT6, SHT7, SHT15	0.000100.00004	0.810.1300
Rikaze	RKZ	3/7	SHT1, SHT2, SHT6, SHT18	0.000060.00002	0.66700.1600
Naqu	NQU	2/7	SHT1, SHT2	0.000030.00002	0.286000.19600
Changdu	CD	2/7	SHT1, SHT15	0.000060.00004	0.286000.19600
Ningqiang	NQ	2/6	SHT1, SHT13	0.000100.00006	0.4000.23700
Baise	BS	4/9	SHT1, SHT9, SHT13, SHT20	0.000190.00005	0.806000.08900
Debao Pony	DB	12/37	SHT1, SHT4, SHT5, SHT7, SHT9, SHT10, SHT11, SHT12, SHT13, SHT14, SHT19, SHT20	0.000160.00002	0.844000.0390
Yunnan Pony	YN	7/8	SHT1, SHT2, SHT4, SHT9, SHT13, SHT16, SHT19	0.000220.00004	0.964000.07700
Zhaotong	ZT	3/7	SHT1, SHT9, SHT11	0.000070.00001	0.714000.12700
Lijiang	LJ	7/34	SHT1, SHT2, SHT4, SHT5, SHT11, SHT13, SHT19	0.000100.00002	0.745000.04900
Tengchong, Yingjiang, Yimen	TC-YJ-YM*	7/25	SHT1, SHT2, SHT4, SHT8, SHT9, SHT13, SHT17	0.000080.00001	0.71000.0600
Guizhou, Jianchang	GZ-JC*	6/13	SHT1, SHT4, SHT5, SHT9, SHT13, SHT19	0.000190.00003	0.872000.05400
Jinjiang	JJ	3/10	SHT4, SHT13, SHT19	0.000180.00006	0.511000.16400
Thoroughbred	TB	2/31	SHT2, SHT3	0.00000580.00001	0.154000.12600
Warm Blood	WB	2/7	SHT2, SHT3	0.000030.00001	0.343000.12800
Arabian	AR	3/15	SHT1, SHT2, SHT3	0.000030.00001	0.343000.12800
Akhal-Teke	AT	2/13	SHT2, SHT3	0.00000.0000	0.000.00

n, the number of haplotypes; N, the number of samples; HT, haplotype; π, nucleotide diversity; h, HT diversity. The geographic distributions of sampled populations are shown in Supplementary Figure S1. *indicates two or three breeds distributed in adjacent places and from which very few samples were collected, so samples of the breeds were merged when their HT diversity and nucleotide diversity were estimated.

### Primer Design for Sequencing ECAY

To investigate Y chromosome diversity in modern horses, primers were designed with Primer V5^1^ based on fragments within the reference sequences of the non-coding regions from 11 BACs of ECAY (Supplementary Table S1). The primers described by Wallner et al. (2013) for detecting the ECAY variants were also used in the present study. Introns of the AMELY and ZFY genes were amplified with previously reported primers (Lau et al., 2009; Supplementary Table S1). To test the ECAY-specific amplifications of these primers, three DNA samples from female horses and ddH_2_O were used as controls in all amplifications of ECAY fragments. Primers that generated target products only in male horses were selected.

### Genotyping and Sequencing ECAY

To detect ECAY nucleotide variants, 96 samples from 8 indigenous Chinese horse populations [including DB (36), BS (3), YN (12), LJ (19), TC (14), NQ (4), CD (4), and RKZ (4)] were initially screened by sequencing DNA fragments amplified from ECAY. MassARRAY was performed by Bioyong Tech (Beijing, China) to genotype all horse samples in our study based on the information from confirmed mutant loci on ECAY and the 52 SNVs (single-nucleotide variants) reported in a previous study (Wallner et al., 2017). Six samples sequenced in the initial step were also genotyped using MassARRAY as controls to assess the accuracy of genotyping. In the phylogenetic study, the *Przewalski’s* horse, and domestic horses were split with 251 SNVs, which were reported by Wallner et al. (2017). PCRs for amplifying and screening ECAY fragments were performed in a 20 μl total reaction mixture containing 1 μM of each primer, 200 μM of each dNTP, 2 μl of 10 × reaction buffer containing 15 mM MgCl_2_, 0.5 unit of Taq polymerase, and 20–30 ng of genomic DNA as a template. Amplifications were conducted in a GeneAmp PCR 9700 thermal cycler (Applied Biosystems, Foster City, CA, United States). The PCR thermal cycling conditions were as follows: 94°C for 3 min, followed by 34 cycles at 94°C for 30 s, annealing at 53–62°C for 30 s, 72°C for 30–90 s, and a final extension step at 72°C for 10 min. PCR products from two samples were mixed and sequenced by BGI Tech (Beijing, China). To confirm the candidate mutations, PCR products of the genomic DNA from each horse from the pool were resequenced for both forward and reverse strands. Amplifications with female horse DNA and ddH_2_O were conducted for each PCR run as negative and blank controls.

### Copy Number Variations Detected With qPCR

Quantitative PCR was applied to determine the CNVs of 11 loci in ECAY fragments from the 283 male samples mentioned above, which consisted of 217 Chinese indigenous horses, 65 foreign horses, and a *Przewalski’s* horse. The primers used for qPCR are shown in Supplementary Table S3. GAPDH, which has two copies on autosomes, was used as a reference gene for normalization. On each plate, we set up wells for standard curve samples, a calibrator, and a negative control. All the test samples were run in triplicate to ensure accuracy. If CT values of same sample differ by 0.3, this sample needed to retest. qPCRs for amplifying were performed in a 20 μl reaction mixture including 10 μl of SYBR Green RealMaster Mix (TIANGEN BIOTECH, Beijing), 0.6 μl of primers (10 pmol/μl), 7.8 μl ddH20, and 1 μl of DNA template (5 ng/μl). Amplifications were conducted in a CFX96 Real-Time System thermal cycler (Bio-Rad, United States). The PCR thermal cycling conditions were as follows: 95°C for 15 min, followed by 39 cycles at 95°C for 10 s, annealing at 56–60°C for 20 s, 72°C for 30 s, and the melting curve was then generated by taking fluorescent measurements every 0.5°C from 65°C until 95°C. The CNVs were determined with the methods described by Hamilton et al. (2009).

### Determination of Allele-Specific Copy Number at Loci in Repeated Fragments

The allele-specific copy number at 6 loci in the repeated ECAY fragments of 93 male samples harboring a specific haplotype (SHT1) defined with Y-chromosome SNPs was determined by pyrosequencing (Langaee et al., 2015). Primers for pyrosequencing were designed by PyroMark Assay Design 2.0 (Supplementary Table S4). PCRs were performed in a 50 μl total reaction mixture containing 50 pM of each primer, 10 mM of each dNTP, 10 μl of 5 × reaction buffer (KAPA), and 20 ng of genomic DNA template. The PCR thermal cycling conditions were as follows: 95°C for 3 min, followed by 40 cycles at 94°C for 30 s, annealing at 55°C for 30 s, 72°C for 1 min, and a final extension step at 72°C for 7 min. Pyrosequencing was conducted in a PyroMark Q96 ID with 40 μl PCR product according to the manufacture’s recommendation (QIAGEN). The relative ratios of two variants were calculated by software Pyro Q-CpG. In order to obtain the ratios of base variants (RBV; which determine the allele-specific copy number), based on MassARRAY results (Supplementary Figure S2), all heterozygotes of 103, 1, 17, and 9 were tested by pyrosequencing. In locus 165 and 102, samples with the same slope were sampled (the same slopes mean that the samples have the same RBV), and samples with inconsistent slopes were all tested by pyrosequencing.

### Population Structure

Genetic variability of ECAY, including HT diversity (h), and nucleotide diversity (π), were quantified by DnaSP v5.0 software (Librado and Rozas, 2009). The parameters were set as “Nucleotide sequence: DNA; Genomic State: Haploid; and Chromosomal Location: Y Chromosome.”

### Phylogenetic Reconstruction

Network v5010 was used to calculate median-joining (MJN) networks based on ECAY sequences^2^. The network calculation was performed as follows: Epsilon (0) was chosen, and the MP calculation option was used to remove excessive links and median vectors. Other parameters were left at the default settings. Unweighted pair-group method with arithmetic means (UPGMA) was employed to calculate the genetic distance within and between populations. Phylogenctic tree was constructed with software Mega5.2 and Poptree.

## Results

### Molecular Diversity Revealed With SNPs in ECAY

In the present study, DNA variants from a total of 53 kb of male-specific Y chromosome sequences were screened across 96 indigenous Chinese horses by sequencing with both known and newly designed primers (Supplementary Table S1), and 14 segregating sites (Supplementary Table S2) were identified. Subsequently, 235 indigenous Chinese male horses and 66 stallions of foreign breeds (Supplementary Table S5) were successfully genotyped with MassARRAY for the alleles of all 14 identified sites. Seven of the 14 segregating sites were in the fragments amplified with the three primer pairs Y-45288, Y-45701/997, and Y-50869 (which are abbreviated as Y288, Y997, and Y869 in the present study, respectively) described by Wallner et al. (2013) and are shown in Supplementary Table S2. Within the 7 sites, 4 (S/N 8, 9, 13, and 14 in Supplementary Table S2) were newly identified in indigenous Chinese horse populations studied in the present study, and the other 3 sites (S/N 10, 11, and 12 in Supplementary Table S2) were reported previously by Wallner et al. (2013). The remainders of the segregating sites were found with newly designed primers and are reported first in the present study.

Single-nucleotide polymorphisms were also observed in amelogenin genes (Y-linked; AMELY), for which no polymorphism was detected in extant horses in previous studies (Lindgren et al., 2004; Lippold et al., 2011). In the present study, 235 Chinese horses were successfully genotyped for AMELY gene alleles. The results showed that 9 samples of Chinese indigenous horses carry allele C of the AMELY gene (simplified as AMELY-C allele), whereas the remaining 225 horses (96% of all successfully genotyped samples at this locus) are all allele T carriers. The 9 C-type horses were from populations in South China, including GZ, DB, YN, LJ, JJ, and BS (the full names of Chinese indigenous horse breeds and their locations are shown in Supplementary Figure S1).

The ECAY nucleotide (π_*Y*_) and HT diversity (h_*Y*_) were estimated at 5.0 × 10^–5^ ± 2.0 × 10^–5^ and 0.818 ± 0.019, calculated with polymorphisms at 14 segregating sites of the 235 Chinese indigenous horses, respectively. In indigenous Chinese horse populations, higher π_*Y*_ and h_*Y*_ values were found for IMG, YN, DB, JJ, GZ, BS, and several other breeds of southwestern China (Table 1), almost all of which were sampled from the mountain populations.

### ECAY Haplogroups Defined by 14 Segregating Sites

**FIGURE 1:**
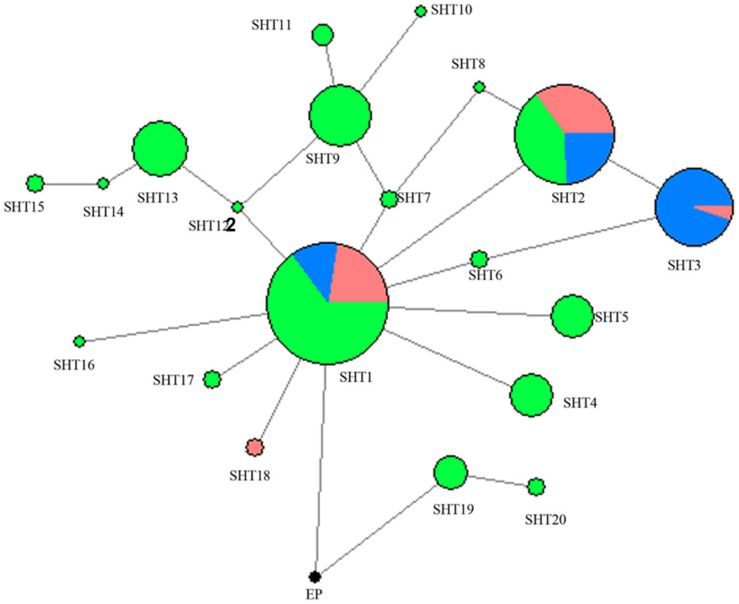
MJN network was constructed using 14 ECAY segregating sites. Circle size was proportional to the haplotype frequency. Blue indicates introduced breeds; green, southern horses; red, northern horses; and black, *Przewalski’s* horse.

A network was constructed with the 14 segregating sites from all of the 302 studied horses in the present study (Figure 1). A total of 21 HTs formed and *Przewalski’s* horse harbors a unique haplotype distinct from the 20 other paternal lines of domestic horses. SHT1 and SHT2 are the two most prominent HTs and found in 28.15% and 16.56% of the studied horses, respectively. Most foreign horses (57.58%) carried SHT3. The horses from South China carried 18 of the 20 HTs, of which 16 HTs were detected only in indigenous horses of South China. SHT19 and SHT20 are the most ancient HTs which were directly rooted from *Przewalski’s* horse, and they were exclusively harbored by the southern Chinese indigenous horses carrying the ancient C allele at the polymorphic site in intron 2 of the AMELY gene.

**FIGURE 2:**
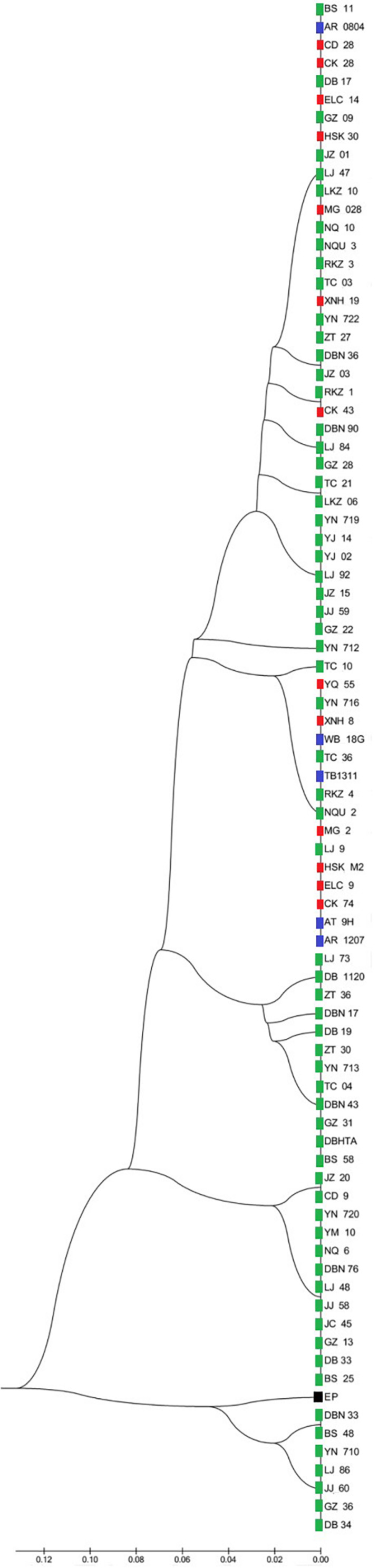
UPGMA tree for all individuals constructed with 14 Y-chromosome SNPs. Individuals from the same breed clustered in one clade were represented by only one sample. Blue indicates introduced breeds; green, southern horses; red, northern horses; and black, *Przewalski’s* horse. Specific sample information and sample locations of distribution are provided in Supplementary Table S5 and Supplementary Figure S1.

**FIGURE 3:**
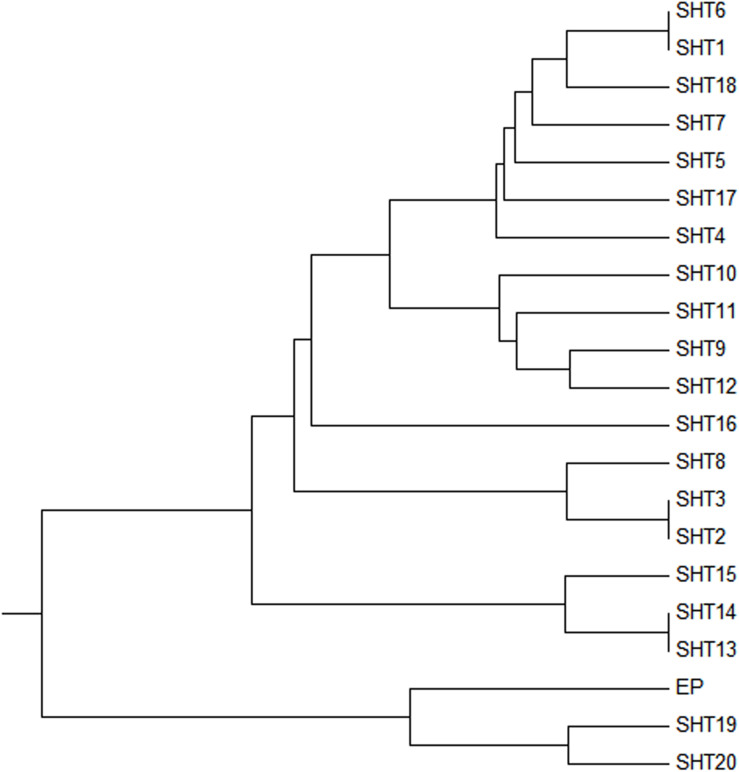
UPMGA clustering dendrogram based on DNA genetic distance among 21 haplotypes formed with 14 ECAY segregating sites.

Two UPMGA clustering dendrograms were constructed with the 14 ECAY segregating sites using Mega 5.2 and exhibit the phylogenetic relationship of the studied populations and the ECAY HTs (Figures 2, 3). The results shown in the dendrogram of the breeds were consistent with those from the network (Figure 1), and horse breeds from South China clustered closely with *Przewalski’s* horse, indicating that some ancient patrilineal lines are preserved in these populations. The clades in the dendrogram of HTs were also in accordance with the history of horse breeding. For example, the three HTs SHT1, SHT2, and SHT3 were found mainly in modern foreign horse breeds, which showed a recent divergence time, while SHT19, SHT20, and most other HTs harbored by horses from South China have a deeper split, which indicates more ancient paternal lines.

### ECAY Phylogenetic Analysis With Merged Data

**FIGURE 4:**
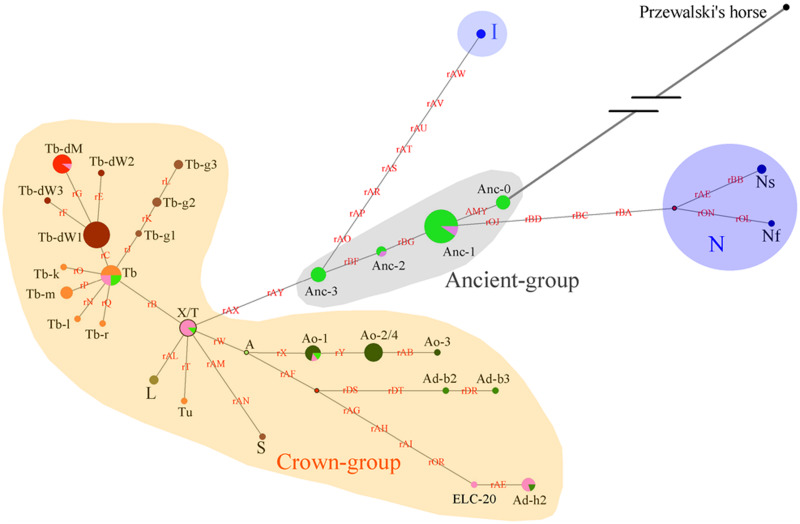
Network constructed with 51 Y-chromosome segregating sites. Brown characters on the lines indicate mutations on single ECAY genes/fragments copies. Black characters near to the pie charts represent the different breeds’ clusters. Crown-group was firstly defined by Wallner et al. (2017) and most of the horse breeds from Europe in our study are included in this group. Sizes of the pie charts are proportional to number of individuals clustered in them. Green and pink pies in Ancient-group refer to horse breeds from South and North China, respectively. Red dots are median vectors.

As the DNA samples of both indigenous Chinese horses in the present study and European horses in the study conducted by Wallner et al. (2013) were genotyped with the same 52 SNP panels, and the C allele variants of AMELY were found only in Chinese horses, the two groups of studied horses were comparable with regard to the data obtained in terms of the SNP panels and known polymorphic sites. An HT network was constructed with the polymorphic data from the 51 segregating sites (genotyping for the other two sites of the 53 polymorphic loci failed for most of individuals, and their data were not used in this analysis; Supplementary Table S6), which contained horses from both the present and previous studies (Wallner et al., 2013; Figure 4). Thirty-eight domestic horse HTs and 1 *Przewalski’s* horse haplotype (Supplementary Table S6) were included in the network.

The network was constructed with merged data from the present study and a previous study (Wallner et al., 2017). The yellow shaded Crown-group, and the blue shaded I and N groups were first described by Wallner et al. (2017). The gray shaded haplogroup was found in the present study and named as Ancient-group, which consisted of 4 HTs: Anc-0, Anc-1, Anc-2, and Anc-3. The colors indicate different horse populations: light green, horses from South China; pink, horses from North China; light blue, Iceland horses; deep blue, horses from northern Europe; deep green, AR or horses with Arabian ancestry; brown, TB, and breeds derived from Thoroughbred stallions; orange, foreign horses sampled in the present study.

Four haplogroups were determined from the 51 segregating sites. The most ancient haplogroup, the Ancient-group, was derived from *Przewalski’s* horse, exclusively includes indigenous Chinese horses, and the majority of stallions from China were distributed in this group. In the haplogroup, Anc-0, rooted directly from *Przewalski’s* horse, is the most ancient haplotype. Three other haplogroups were derived from the ancient group. The largest haplogroup, the crown group, named by Wallner et al. (2017), mainly consisted of AR and horse breeds sired by stallions of AR as their ancestors, such as TB, Warmbloods, and other European horses from Thoroughbred lines. The Crown-group also included a few indigenous Chinese horses. The haplogroup N group consisted of Fjord, Shetland Pony, and Coldblood, which are from northern Europe. Horses from Iceland formed haplogroup I.

**FIGURE 5:**
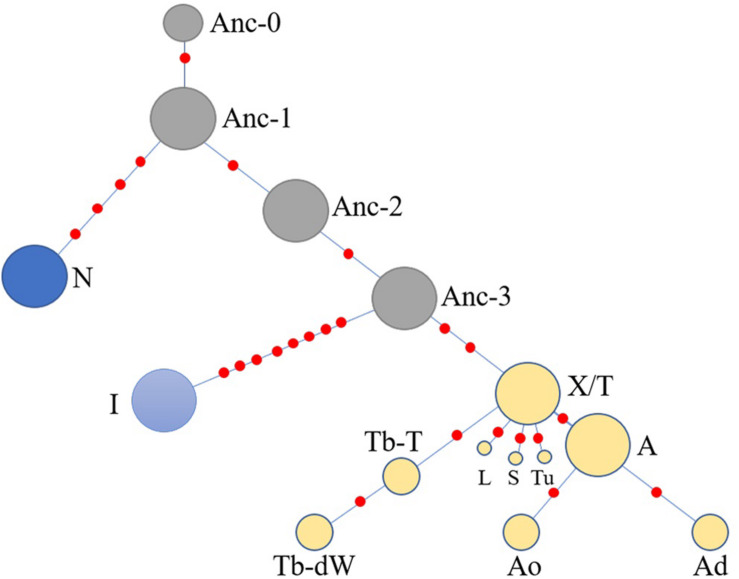
Simplified network illustrating relationships among main haplotypes. The haplotypes are correspondent to those in Figure 4.

Another simplified network was constructed based on the haplotype data to illustrate of the relationships between HTs (Figure 5). The Anc-0 in the Ancient-group was the foremost ancestral haplotype, and the other HTs were derived from it (Figure 4). As shown in Figure 4. The Anc-1, Anc-2, and Anc-3 HTs, harbored by Chinese horses, arose from Anc-0 through one or more mutations. The HTs in haplogroups N and I and the Crown-groups were derived from Anc-1 and Anc-3, respectively. It is worth noting that the ancestral Anc-0 haplotype consisted of five indigenous Chinese horses carrying the C allele at the polymorphic site in intron 2 of the AMELY gene. Previous studies showed that only donkeys, *Przewalski’s* horse, and ancient horses harbored the C allele at the locus, while all modern horses exclusively carried the T allele. Our study also confirmed that the majority of extant horses carry the T allele, but we did find that a few indigenous horses from South China are C allele carriers. The network results also indicated that horses with the C allele belong to the ancestral group.

### Copy Number Variations of Studied Y-Chromosome Sites

According to the genotyping results, 11 Y-chromosome fragments, most of which carry double base-calling SNPs, were determined for CNV with qPCR techniques (Supplementary Table S7). The results showed that the copy numbers vary greatly among loci and individuals. Loci 9, 17, 102, 103, and 165 have more copies than the others, and loci 9 and 17 also showed greater copy number variations (Tables 2, 3).

**TABLE 2:** Median number and range of copy variations of the studied horse populations at 11 loci.

	**Loci**										
**Breed**	**165**	**17**	**102**	**103**	**9**	**14**	**1**	**12**	**132**	**133**	**4**
NC	5 (5 ∼ 10)	5 (3 ∼ 8)	20 (10 ∼ 20)	5	5 (2 ∼ 8)	1	1	2	1	1	1
SC	5 (5 ∼ 10)	5 (3 ∼ 10)	10 (10 ∼ 20)	5	5 (2 ∼ 10)	1	1 (1 ∼ 2)	2	1	1	1
NSC	5	5 (5 ∼ 6)	10 (10 ∼ 20)	5	5 (5 ∼ 6)	1	1	2	1	1	1
IN	5 (5 ∼ 10)	6 (3 ∼ 10)	20 (10 ∼ 20)	5	5 (4 ∼ 10)	1	1	2	1	1	1
EP	10	8	20	5	5	1	1	2	1	1	1

**TABLE 3:** Polymorphism information of CNVs in five studied populations at 11 loci.

**Locus**	**165**	**17**	**102**	**103**	**9**	**14**	**1**	**12**	**132**	**133**	**4**
NC	0.11	0.39	0.37	0	0.34	0	0	0	0	0	0
SC	0.06	0.42	0.35	0	0.37	0	0.14	0	0	0	0
NSC	0	0.30	0.36	0	0.36	0	0	0	0	0	0
IN	0.36	0.47	0.31	0	0.31	0	0	0	0	0	0
EP	0	0	0	0	0	0	0	0	0	0	0

### Haplotypes Defined With SNPs, CNVs, and AS-CNVs

Detailed patrilineal lines were revealed with both Y-chromosome SNPs and the CNVs of homozygous samples (Supplementary Table S7). The analysis showed that the same HTs defined with SNPs may contain different paternal lines when the CNVs were considered. In addition, CNVs of the heterozygous samples were also studied. As mentioned above, six polymorphic Y-chromosome loci (loci 1, 9, 17, 102, 103, and 165) showed double base calls, which are attributed to plenty of repeated fragments in the male-specific Y chromosome. To further characterize these special segregating sites, we determined the AS-CNVs of heterozygous samples by pyrosequencing. The results showed that some loci have a high level of polymorphism at the AS-CNV level, such as loci 9 and 17, each of which has 6 combination patterns of the two alleles in the AS-CNVs of heterozygous samples (Table 4).

**TABLE 4:** Combination patterns of AS-CNVs in the heterozygous samples at the six segregating sites in repeated fragments.*

**Locus**	**165**	**103**	**1**	**17**	**9**	**102**

**base variants**	**G:A**	**C:A**	**A:G**	**G:A**	**G:A**	**G:T**
ratios	4:1	4:1	1:1	1:1	1:1	3:2
				1:4	1:2	4:1
				2:1	1:4	7:3
				2:3	2:1	
				3:2	2:3	
				4:1	3:2	

*The combination patterns of AS-CNVs of the two alleles at each site are indicated by the ratio of the copy numbers of the two alleles [ratios of base variants (RBV)], which were determined with pyrosequencing.

Our results showed that the diversity of the patrilineal lines of the heterozygous samples could be represented with AS-CNVs. Locus 9 is presented an example to show how the different patrilineal lines defined with AS-CNVs derived from SNP backbones. At locus 9, the “G” mutated to “A” or “GA” (in the repeated fragment), and the patrilineal lines of individuals heterozygous at the site could be distinguished from each other by examining the combination patterns of the AS-CNVs of the two alleles (G and A). For example, “G3A2” and “G2A3” [the abbreviations of HTs for the heterozygous samples carrying 3 copies of the G allele plus 2 copies of the A allele (G3A2), and 2 copies of the G allele plus 3 copies of the A allele (G2A3), respectively] come from different paternal lines, though they have the same heterozygous genotype and the same total copy number. A phylogenetic tree was constructed using the studied samples in our study with the variants of SNPs, CNVs, and AS-CNVs (defined with RBV) data determined in the present study (Supplementary Figure S3 and Supplementary Table S8). And the tree is accordant with the results showed in Figure 3. It is showed in Supplementary Figure S3 that the HTs derived from ECAY SNPs contains different clusters which could be distinguished by CNVs, while some clusters of heterozygous samples defined with SNPs and CNVs could be further divided into sub-clusters with AS-CNVs.

## Discussion

### Phylogenetic Studies on Patrilineal Resources of Chinese and Overseas Horse Populations

A single base call is expected at every nucleotide position on the haploid Y chromosome. However, some of the loci assessed in this study exhibited double base calls, similar to diploid variation, which resulted from duplications in the male-specific Y chromosome (Supplementary Table S2). Except the pseudoautosomal regions, the Y non-recombining region does not exchange genetic material with the X chromosome. Hence, mutations accumulate and are not repaired on the Y chromosome, and muti-copy genes are common in human and equine Y chromosome (Subramanian, 2001; Janecka et al., 2018). The 14 segregating sites detected by sequencing in the present study were used to estimate the ECAY nucleotide and haplotype diversity instead of the data from the 51 merged polymorphic sites because the directly genotyped data may lead to underestimation of diversity. The 66 studied foreign horses were also genotyped with MassARRAY for the 14 segregating sites, and they had higher ECAY π_*Y*_ values than those reported by Wallner et al. (π = 3.71 × 10^–6^; Wallner et al., 2013), but they were still significantly lower than those of Chinese horses. These results showed that indigenous Chinese horses preserved more Y chromosome diversity than European horses. DNA data from 9 ancient horses [consisting of 8 ancient wild horses and a 2,800-year-old domestic horse described in a previous study (Lippold et al., 2011)] exclusively contained allele C in the second intron of the AMELY gene, which is in contrast with the uniform allele T identified in all extant horses previously studied (Lindgren et al., 2004; Lippold et al., 2011). This finding is in accordance with the fact that the majority of indigenous Chinese horse breeds have not been subjected to intense selection. Both the MJN network and UPMGA clustering dendrograms derived from the 14 segregating sites indicated that the prominent HTs harbored by indigenous Chinese horses are distinct from those of foreign horses. In addition, some indigenous Chinese horses carried ancestral ECAY HTs, especially horses from South China, which carry most of the private Y-chromosome HTs, and 9 of them harboring the ancient AMELY-C allele forms the ancient haplotype SHT19 and SHT20.

When we used a merged data set derived from the 51 segregating sites that consisted of 50 SNVs reported previously (Wallner et al., 2017) and the SNP in the AMELY gene found in the present study, the results also showed that there are some ancestral HTs distributed among indigenous populations in China. There are four Y-chromosome HTs exclusive to indigenous Chinese horses in the network (Figure 4), namely, Anc-0 (simplified from Ancient-0), Anc-1, Anc-2, and Anc-3. Most individuals carrying the ancient HTs are from South China. The most-ancestral haplotype is Anc-0, which is found solely in indigenous horses from South China harboring the ancient AMELY-C allele identical to that in ancient and *Przewalski’s* horses. Our results also showed that the N, I, and the so-called ALST HTs mainly come from the Shetland Pony and Norwegian Fjord horses (N), Iceland horse (I), and Lipizzaner, Sorraia, and other modern horse breeds related to Arabians and Thoroughbreds (ALST), respectively, as previously reported by Wallner et al. (2017). In our results, the haplotype N arose from Anc-1, while the I and ALST HTs are derived from Anc-3 through two or more step mutations.

As Wallner et al. estimated, Asian male horses have the most ancient paternal lines that diverged more than 5,000 years ago (Felkel et al., 2018). In their study, only some horses from North China were included, in which a Mongolian and a Yakutian horse harbored the ancestral HTs. In the present study, the results showed that horses of southwestern China carrying the AMELY-C variant exhibited the oldest HTs (Anc-0), and the HTs in northern horses were derived from Anc-0. The results suggest that there are some most ancient patrilines in southwestern China. Indigenous Chinese horse populations have largely preserved their genetic diversity because of no intensive breeding strategies imposed on them (Cai et al., 2009). Specifically, the people in mountainous areas in southern China prefer indigenous horses because of their great adaptation to the local environment and their ability to function as pack animals in the mountains. Therefore, their autochthonous genetic variants are preserved. Because the genetic characteristics of ancestral horses are better preserved in indigenous populations, these horses can be reasonably regarded as the remains of the earliest domestic horse patrilines in extant domestic horse populations. Though hundreds of Chinese local male horses were investigated and the Y chromosomal data of European horses from the previous study were jointly analyzed in our study, it is still necessary that a large panel of samples consisted of global samples, especially foreign indigenous male horses, should be used to further validate our phylogenetic results and make a more detailed illustration on the evolution of male horses.

The replacement of the AMELY allele C in ancient wild with allele T of domestic horses, found in extant horses, was described in both previous studies and the present study, indicating that a selective sweep occurred. In the present study, though both allele C, and allele T were detected, most allele C carriers were clustered in the SHT19 and SHT20 haplogroups (Figure 2), suggesting a close genetic relationship among allele C carriers. Based on DNA data acquired from both ancient and extant horses, it can be deduced that a selective sweep related to the AMELY locus occurred during domestication and has lasted to the present day, possibly caused by advantageous mutations linked with the AMELY gene on the Y chromosome. As shown in previous studies and our results, polymorphism of AMELY was completely lost in extant European horse populations compared with indigenous Chinese horses (Lindgren et al., 2004; Ling et al., 2010; Lippold et al., 2011; Rafeie et al., 2011; Wallner et al., 2013), which indicates that the selective sweep was accelerated by intensive breeding activities. Our results suggested that the selective sweep affected the diversity of the ECAY loci in a slow but global way, in contrast to artificial selection or genetic introgression involved in intensive breeding practices, which usually have significant impacts on populations in relatively restricted geographical areas but occur in a rapid manner and could substantially change the genetic structure of populations over several generations. The rare alleles of the AMELY gene identified in the present study suggest that horse populations in South China are at the end of the selective sweep, both geographically and historically. And priority of conservation should be given to these horse populations.

### Fine Discrimination of Horse Patrilineal Lines With the Novel Y-Chromosome Markers

In most livestock, Y chromosomes harbored by paternal lines usually lack polymorphisms at DNA sequence level, which is opposite to mtDNA, which abounds with sequence variation, especially in the highly varying regions of the mitochondrial sequence. The extremely low diversity of horse Y chromosomes has also been reported in several previous studies (Levine, 1999; Cunningham et al., 2001; Wallner et al., 2004; Lippold et al., 2011). In the present study, 14 segregating sites were identified, and 11 of them were novel markers first identified in the present study, which add valuable markers for the genetic study of horse paternal lines. However, generally the nucleotide variants on horse Y chromosomes are still very insufficient (Wallner et al., 2017). The limited number of polymorphic sites on the Y chromosome leads to a low phylogenetic resolution of horse patrilineages.

It is well known that the Y chromosome of mammalian animals is rich in repeated sequences, which has also been reported in previous studies on horses (Wallner et al., 2017). Eleven of the 14 segregating sites detected in the present study were proved to be located on repeated sequences, which also indicate the presence of many repeats in the Y chromosome. CNVs could serve as genetic markers for phylogenetic studies (Pierce et al., 2018; Zhang et al., 2019), so we explored the diversity of the CNVs of ECAY, and used them to conduct phylogenetic analysis. qPCR is sensitive and error may be caused by random factors. To overcome it, three repeats were set for each sample, and the samples were determined again if the values of the repeats varied remarkably. We analyzed patrilineal lines derived from the SNP backbone and the CNVs of repeated sequences. The results indicated that even paternal lines harboring the same allele at the SNP site could be further classified into different sub-lines according to the polymorphisms of the copy numbers in the fragments where the SNP site is located. The jointly analysis of the three types of markers (Supplementary Figure S3 and Supplementary Table S8) also verified our results derived merely from SNPs data (Figure 3).

The double base-calling sites caused by the repetitions in Y-chromosome sequences, which are similar to the heterozygous genotypes of autosomes, not only show a polymorphism of the genotypes that is similar to the two-allele polymorphic loci of autosomes, but also exhibit the polymorphisms caused by copy number variances (Langaee et al., 2015). The ratio of the copy numbers of the two alleles at heterozygous sites of the Y chromosome is not necessarily 1:1, a situation that is usually found at the heterozygous loci of autosomes. Our results showed that some combination patterns of AS-CNVs are highly polymorphic, such as at locus 17, which has 6 combination patterns of AS-CNVs in the studied horse populations. The various combination patterns of AS-CNVs in ECAY are caused by asymmetric repetition of the fragments that contain distinct alleles. Individuals carrying the same heterozygous genotype and the same copy number of the repeated fragments can still be distinguished from each other if they have different combination patterns of AS-CNVs. More detailed patrilineal lines could be revealed by jointly using the Y-chromosome SNPs, CNVs, and AS-CNVs. The novel Y-chromosome marker systems consisting of the three types of markers could lead to higher resolution of horse patrilineal lines and provide new methods for studying and assessing genetic diversity of male horses.

SNPs and CNVs are slow and rapidly evolving polymorphic markers, having an average mutation frequency of 10^–8^ and 1.7 × 10^–6^–1.0 × 10^–4^ per generation in humans, respectively (Redon et al., 2006; Freire-Aradas et al., 2012). The combined use of the slow and rapidly evolving polymorphic markers on the horse Y chromosome could make it possible to finely distinguish among horse paternal lineages and greatly facilitate the study of genetic diversity and assession of paternal diversity, which are essential for evolution and immigration of horse paternal lineages, and making rational conservation plans for the rare patrilines. As males of mammalian animals have usually been subjected to relatively high natural or artificial selection, there are fewer nucleotide variants identified in male populations than in female groups. The strategies of joint use of the slow and rapidly evolving markers in this study can also serve as valuable reference for the patrilineal genetic and evolutionary studies in other mammalian species.

## Conclusion

In conclusion, our Y-chromosome DNA data indicated that relatively high ECAY diversity and ancient paternal lines were preserved in indigenous Chinese horses. The Y-chromosome marker system developed in the present study provides a new strategy to investigate and assess the diversity of horse patrilineages.

## Author’s Note

Biosecurity issues are not involved in this study.

## Data Availability Statement

Our data has been deposited in European Variation Archive (EVA). Following are their accession information: SNP: PRJEB35322 and CNV: PRJEB37580.

## Ethics Statement

This study was carried out in accordance with the principles of the Basel Declaration and recommendations of Animal Care and Use, and the requirement of the Animal Care and Use Committee of China Agricultural University. The protocol was approved by the Animal Care and Use Committee of China Agricultural University.

## Author Contributions

CZ conceived and designed the study. CW reviewed and improved the experiments design and collected some important samples for the study. SL and YY performed the experiments. SL, YY, and CZ analyzed the data. The manuscript was written by SL, YY, and CZ. All authors contributed to the submitted version of the manuscript.

## Conflict of Interest

The authors declare that the research was conducted in the absence of any commercial or financial relationships that could be construed as a potential conflict of interest.

## Footnotes

1 http://PremierBiosoft.com

2 http://www.fluxus-engineering.com

## Supplementary Material

FIGURE S1 Geographic distributions of sampled Chinese indigenous horse populations.

FIGURE S2 The MassARRAY genotyping results.

FIGURE S3 The merged SNP-CNV-RBV data analysis.

TABLE S1 ECAY-specific Primers for screening SNPs.

TABLE S2 Segregating sites identified in the present study.

TABLE S3 Primers for real-time PCR.

TABLE S4 The information of primers for pyrosequencing.

TABLE S5 The genotyping results of the studied horses at the 14 segregating sites screened in the present study.

TABLE S6 The merged SNP genotyping dataset.

TABLE S7 The CNVs of the DNA fragments containing the 11 segregating sites.

TABLE S8 The merged SNP-CNV-RBV data. The CNVs of each site were divided into two types by median (<median, A; ≥median, B). Letters **(A–G)** represented the Ratios of Base Variant (RBVs).
