# Supplementary figures and images for: Ancient Patrilineal Lines and Relatively High ECAY Diversity Preserved in Indigenous Horses Revealed With Novel Y-Chromosome Markers
Source: Front Genet. 2020 May 21;11:467. doi: 10.3389/fgene.2020.00467 (PMC7253630; doi:10.3389/fgene.2020.00467)

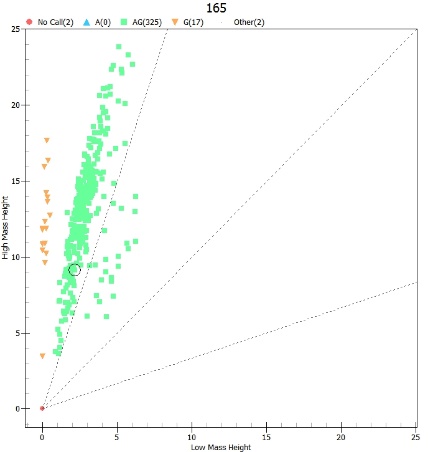

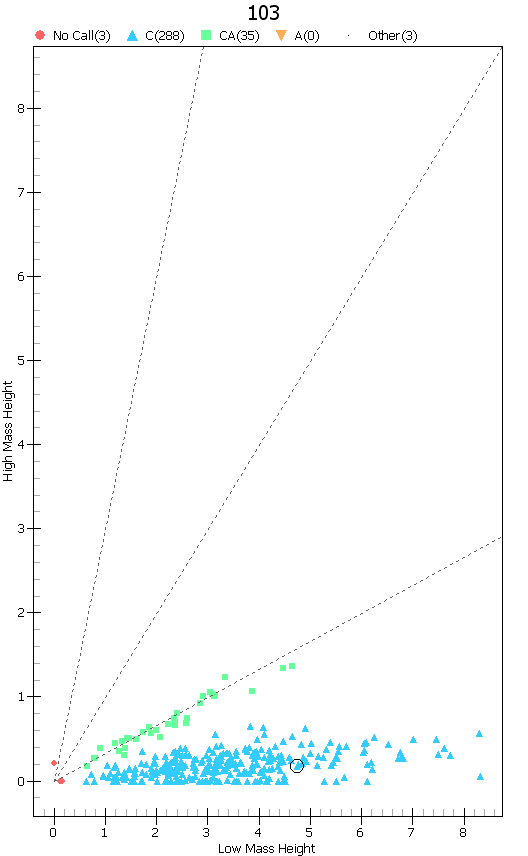

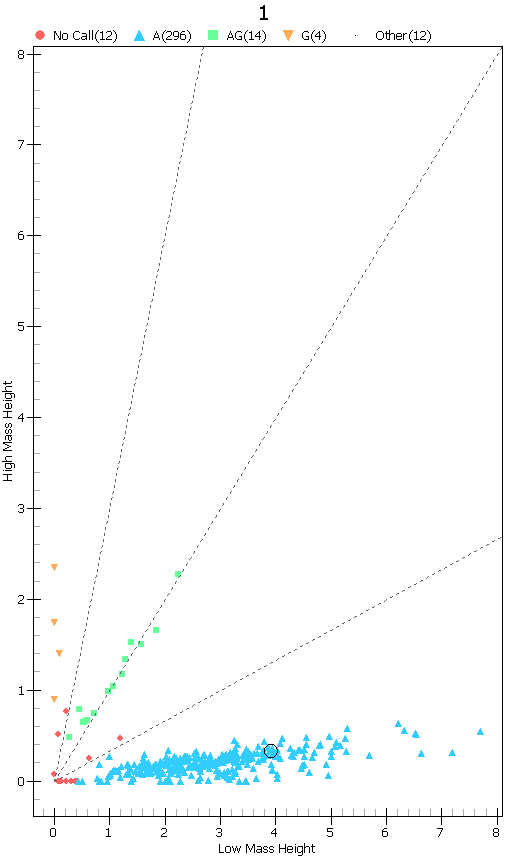

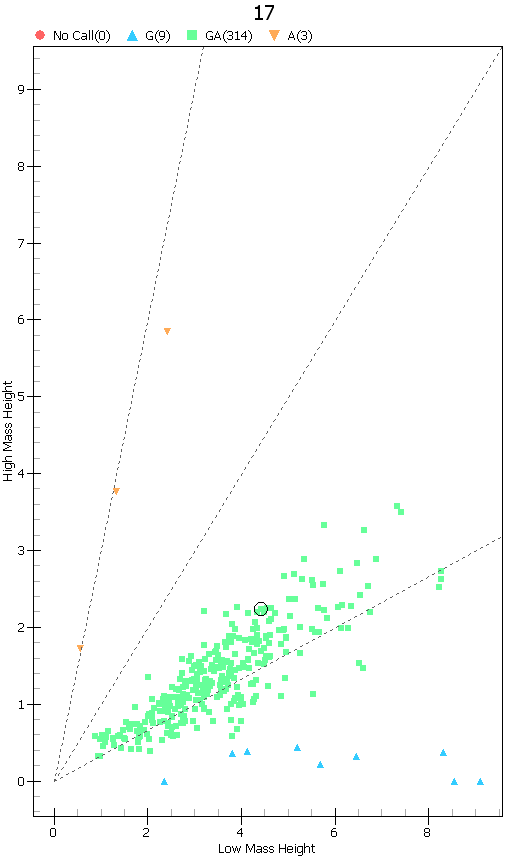

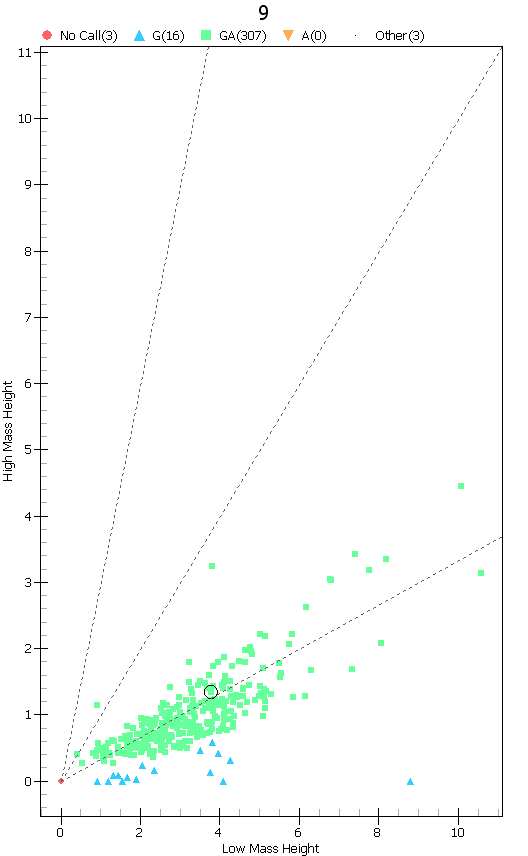

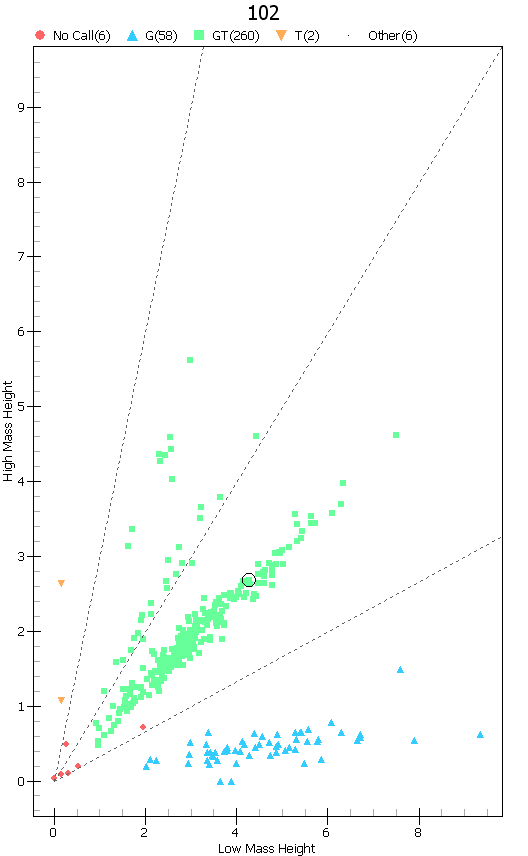

Supplement: FIGURE S1 — Geographic distributions of sampled Chinese indigenous horse populations. [file Data_Sheet_1.ZIP › supplementary materials/Fig S2. The MassARRAY genotyping results.docx]

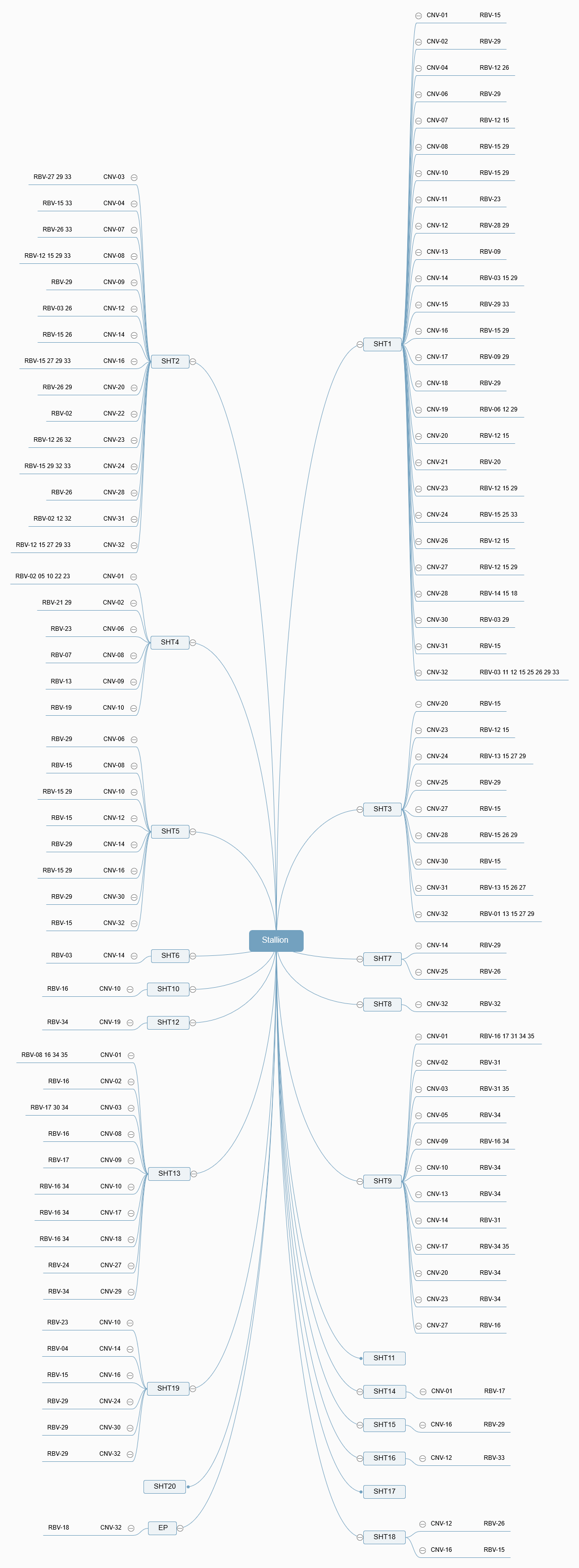

Supplement: FIGURE S1 — Geographic distributions of sampled Chinese indigenous horse populations. [file Data_Sheet_1.ZIP › supplementary materials/Fig S3. The merged SNP-CNV-RBV data analyis.png]
