# Supplementary material for: Ancient Patrilineal Lines and Relatively High ECAY Diversity Preserved in Indigenous Horses Revealed With Novel Y-Chromosome Markers
Source: Front Genet. 2020 May 21;11:467. doi: 10.3389/fgene.2020.00467 (PMC7253630; doi:10.3389/fgene.2020.00467)
Supplement: FIGURE S1 — Geographic distributions of sampled Chinese indigenous horse populations. [file Data_Sheet_1.ZIP › supplementary materials/Fig S1. Geographic distributions of Chinese indigenous horse populations .doc]

**FigS1. Geographic distributions of sampled Chinese indigenous horse populations**

Localities of all samples were characterised by population names. The population abbreviations and their locations are as follows: Mongolian Horse of inter-Mongolia (IMG), Xinihe Horse (XNH), and Elunchun Horse (ELC) from Inner Mongolia Province; Chakouyi Horse (CK) from Gansu Province; Rikaze Horse (RKZ), Jiangzi Horse (JZ), Naqu Horse (NQU), Changdu Horse (CD), and Langkazi Horse (LKZ) from Tibet Province; Kazakh Horse (KZK) and Yanqi Horse (YQ) from Xinjiang Province; Debao Pony (DB) and Baise Horse (BS) from Guangxi Province; Lijiang Horse (LJ), Tengchong Horse (TC), Zhaotong Horse (ZT), Yingjiang Horse (YJ), Yimen Horse (YM), and Yunnan Pony (YN) from Yunnan Province; Jianchang Horse (JC) from Sichuan Province; Guizhou Horse (GZ) from Guizhou Province; Jinjiang Horse (JJ) from Fujian Province; Ningqiang Horse (NQ) from Shanxi Province, respectively.
