# Supplementary material for: Ancient Patrilineal Lines and Relatively High ECAY Diversity Preserved in Indigenous Horses Revealed With Novel Y-Chromosome Markers
Source: Front Genet. 2020 May 21;11:467. doi: 10.3389/fgene.2020.00467 (PMC7253630; doi:10.3389/fgene.2020.00467)
Supplement: FIGURE S1 — Geographic distributions of sampled Chinese indigenous horse populations. [file Data_Sheet_1.ZIP › supplementary materials/Table S1. ECAY-specific Primers for screening SNPs .doc]

**TableS1. ECAY-specific Primers for screening SNPs**

All the new primers were designed with Primer Premier V5.0 ([http://wwww.PremierBiosoft.com](http://wwww.PremierBiosoft.com/)). PCRs were conducted with PCR Mix (provided by HT-Biotech Company, Beijing, China. [http://www.ht-biotech.net](http://www.ht-biotech.net/)).

| BAC or Gene | Accession NO. | Primer | Fragment  Length [bp] | Annealing  Temp.[ °C] | Forward(5’ to 3’) | Reverse(5’ to 3’) |
| --- | --- | --- | --- | --- | --- | --- |
| **clone XX-131N23** | AC218925.1 | N23-2 | 1511 | 60 | GACCAGCCCACAAAATGAG | CTCCCCTCTTAGCCTTTGG |
|  |  | N23-3 | 908 | 60 | AGACGGTTCTGGTAAGTAGTGC | CATCATTGGCTTCAGGGTCTA |
|  |  | N23-4 | 1130 | 60 | CAGAGTTTCTCAGCAGGTC | GATAGAGTATTCTTGGCTGT |
|  |  | N23-5 | 1582 | 60 | GTGCCATAACTCGTGTAAG | GTATTTGCCCTTACCCTAT |
|  |  | N23-6 | 1067 | 60 | ATACAAACCAGCTATCCCACT | CTTCCCTGCTTGACTTGATA |
|  |  | N23-7 | 1528 | 57 | AGAGCCAGTGTTCAGTTAGT | ATCCAGGTATCAATGTAAG |
|  |  | N23-8 | 1674 | 60 | TTTGTATCTTGGCTCTTGTG | CAATAATGAAACAGCGGACT |
|  |  | N23-9 | 976 | 60 | AGACCCATCCTACAGACCTA | TCTTGGCCTGTAGAGTTCTG |
|  |  | N23-10 | 913 | 60 | GGAGGTATCCATTCCGTGTT | TGACACCTGCCTTTGACTG |
|  |  | N23-11* | 906 | 60 | GGCAGACATTCAAAGAGTT | ATGGCTGATGAGTGGAGTA |
| **Y subclone M88** | JX565701.1 | M88-1 | 1210 | 60 | AGGCACATCCTGTTCATAC | CATAAGCAACTGAGAACCC |
|  |  | M88-2 | 1615 | 60 | GTGGGACTGCCGTTATGGT | AAACGCCTCAAACAGCACC |
|  |  | M88-3 | 1675 | 61 | GTGGCAGCACCAATTAGCAT | GGAGGCTTTGCCCTTAGTTT |
| **Y subclone M89** | JX565702.1 | M89-1 | 936 | 60 | ACTTTGCCTAATGTGGGAC | ACTTTGCCTAATGTGGGAC |
|  |  | M89-2* | 1089 | 57 | GGTCCGTAGCCTCTAACTCT | AAGGCTCACCTAGAATAACG |
|  |  | M89-3 | 1584 | 62 | TCATATCACCTAAACCAGGAA | TACACTAGCAAACCAACCC |
| **Y chromosome clone E52** | JX565705.1 | E52-1 | 1589 | 61 | TCACTGGGTGGTCATAGAA | ATCACGGGTGACTGAAGAC |
|  |  | E52-2 | 1271 | 60 | ATGATAATTGGCTGCTTTC | CAGAGGTGATTACAGGTGC |
|  |  | E52-B1 | 1270 | 61 | CCATACCTGGGAACTGAAC | CAGGCAAGTGTCTTATGGA |
|  |  | E52-B2 | 703 | 58 | TGCCTTTGTTTCTTGTGA | AACGCTTACTTCCTTGTC |
|  |  | E52-B3 | 1654 | 58 | TGCTCCTGTATTGTTTGATT | AATAAGCCTTTGTCGGTTC |
|  |  | E52-B4 | 1174 | 60 | TGGCTTCTACCAGGCTATTC | CCCTGATGGCTTAGTGGTTA |
| **Y subclone NO167** | JX565706.1 | 167-2 | 1314 | 60 | ATCCTTCTGGCTGGGCTAC | AGACAGCCCATCGCAACTC |
|  |  | 167-3 | 1348 | 60 | AGCCACAATACCAATACCG | AGATGGGTTAGCACGAGAA |
|  |  | 167-4 | 1555 | 53 | ATGCTTGGCTCTTGGTTAT | TTGTTCACTCTTAGCGTTG |
|  |  | 167-B1* | 798 | 58 | GTTTCTACCGTCTTTCCATT | TTAGACTGACAACTTGGTGG |
|  |  | 167-B2 | 912 | 62 | AATGCTTGGCTCTTGGTTAT | TTATCCCTTCATTCATTTCG |
| **Y subclone M23** | JX565700.1 | YM23-1 | 1275 | 61 | TACGGTTCCCACAACTGCG | AAATCCACCCAGCACTTCA |
|  |  | YM23-2 | 1500 | 62 | ACCCTTAGAGTTTCCTGACA | CTGGCTGACTATCGGTTCTA |
|  |  | YM23-3 | 1241 | 60 | ATTGGATTGCTGCGTGATGG | TGGGCTCTGAAAGGAGTTGGTG |
|  |  | YM23-4 | 1332 | 61 | TTGTTCCTGCTCTTGGTTGC | TATGCTCCTCAGGACTGGTT |
| **Y subclone E3** | JX565703.1 | YE3-1 | 1660 | 62 | CTGGGTGGACTCTGTGATG | AGGGCAACTGATAGACGAG |
|  |  | YE3-2 | 1227 | 55 | TTACCATTCCCTACTTGACT | TAAATATCACGCCATAGGA |
|  |  | YE3-3 | 1467 | 61 | GGCTCCCATCAATCCTTCA | AGGGCATCCTTGTCTCGTT |
|  |  | YE3-4 | 735 | 61 | CTCCCGCTGACTCCTATGAAC | TACTGTAGACTGCTGCCGTTA |
|  |  | YE3-5 | 704 | 61 | TTCTGAAACCGCAAGATAGT | TAGTAGTGCTCCCGAGTGAT |
|  |  | Y288** | 297 | 60 | CCCTCTGCTGAGCATCTAGG | TTGGATGAAAGGGACAGTGA |
|  |  | Y997** | 444 | 60 | CCAACACACGTCAACAGCTC | GGCTTAGGCCACTGATGGTA |
| **Y subclone NO217** | JX565707.1 | YNO217-1* | 995 | 60 | ATGGCACCAAGGATGTCAGT | TGTCAACCCTGATAGAGTGC |
|  |  | YNO217-3 | 1527 | 61 | GGGATAGTTTAGGACGCATTA | TTACATCCTCCACTGAAACC |
|  |  | YNO217-4 | 952 | 61 | GGTTTCAGTGGAGGATGTA | GAATACTTACCCTGTCTACC |
| **Y subclone P41** | JX565709.1 | P41-1-1 | 1091 | 61 | AAGGTTAAGTTGGCGGTCTC | AAATGCTCGTTCTTGTCTGC |
|  |  | P41-1-2 | 1681 | 61 | AAGGTTAAGTTGGCGGTCTC | TCAGCGGAGAAGTAACAGAT |
| **XX-024I23** | AC215855.2 | H2 | 1851 | 55.8 | AAGCTGGTATCTACATTTCGG | CTGTCTAGCCTCCTCTATTCG |
|  |  | H15 | 1841 | 61 | TGTTGGATCTGAAACCGTGTA | CTATGAAAGGGAGCAATAGTCA |
|  |  | H19 | ~2000 | 59.8 | TGTATTGACGGATTTCGGACTA | TGTGGCTGAGAATGTAAGAGG |
|  |  | H26 | ~2000 | 61 | CCGCAGAAGAAGAGAGACAA | TCCTGAGGGATGACAGTGGT |
|  |  | Pre34 | 1115 | 54.7 | CTGGGTTACAAACGACTGC | GTAAGCTGGTACTGGTAGTGA |
|  |  | Y345** | 246 | 60 | CCTCCGGCCTTTATGTCTTAG | TTGGGCTGCAGTATACAACG |
| **ZFY** | FJ222532.1 | A2** | 403 | 55 | TAGCAGTTCAAGGATTGCATA | GGACAAATTACACAGTATTCTT |
| **AMELY** | AB091794 | In2-2 | 1225 | 60 | CTCAATTCAACACCCTTACT | GAAACTGGTAGTGGGAACTG |
| **YE17** | JX646955.1 | Y869** | 264 | 60 | GGCCTAAGTTGTTCGCAGAG | TGACTGGTGGTGTCCAGTGT |

* The Primers did not work well and were not used in the consequent study.

** The primers, and PCR conditions described in previous studies were used in the present study (Wallner et al., 2013; Lau et al., 2009).
