# Supplementary material for: Ancient Patrilineal Lines and Relatively High ECAY Diversity Preserved in Indigenous Horses Revealed With Novel Y-Chromosome Markers
Source: Front Genet. 2020 May 21;11:467. doi: 10.3389/fgene.2020.00467 (PMC7253630; doi:10.3389/fgene.2020.00467)
Supplement: FIGURE S1 — Geographic distributions of sampled Chinese indigenous horse populations. [file Data_Sheet_1.ZIP › supplementary materials/Table S2. Segregating sites identified in the present study.doc]

**Table****S2.** Segregating sites identified in the present study*

| Serial number | **1** | **2** | **3** | **4** | **5** | **6** | **7** | **8**^#^ | **9**^#^ | **10**^#^ | **11**^#^ | **12**^#^ | **13**^#^ | **14** |
| --- | --- | --- | --- | --- | --- | --- | --- | --- | --- | --- | --- | --- | --- | --- |
| Site No. | **165** | **17** | **102** | **103** | **9** | **14** | **1** | **111** | | **112** | **12** | **132** | **133** | **4** |
| Reference | **G** | **A** | **G** | **C** | **G** | **T** | **A** | **GT** | | **T** | **T** | **G** | **G** | **C** |
| Alternative | **A** | **G** | **T** | **A** | **A** | **C** | **G** | **CC** | | **-** | **A** | **T** | **A** | **G** |
| Basecalls detected^*^ | **G** | **A** | **G** | **C** | **G** | **T** | **A** | **GT**  **CC**  **CC.GT** | | **T** | **T** | **G** | **G** | **C** |
|  | **AG** | **AG** | **GT** | **AC** | **GA** | **C** | **AG** |  |  | **-** | **A** | **T** | **A** | **G** |
|  |  | **G** |  |  | **A** |  |  |  |  |  |  |  |  |  |
| BAC | AC215855.2 | | AC215855.2 | | AC215855.2 | AB091794 | AC218925.1 | JX565703.1 | | | JX646955.1 | JX565703.1 | | JX565700.1 |
| Position in  BAC | 34528 bp  35202 bp | | 32894 bp  33164 bp | | 9497 bp | 2614 bp | 33601bp | 11490 bp  11491 bp  11515 bp | | | 1277 bp | 12023 bp  12256 bp | | 9610 bp |
| Primer used^**^ | Pre34 | | H15 | | H2 | IN2-2 | N23-5 | Y288 | | | Y869 | Y997 | | M23-4 |

*Hyphens indicate indels; Basecalls detected, the basecalls detected in the horses genotyped in the present study; Primer used, the primers used for amplifying the segregating sites. Detailed information of the primers is shown in TableS1. Single basecalls are expected at every nucleotide position of the haploid Y-chromosome. However, some loci in the present study have double basecalls resembling diploid variants due to duplication of the DNA segment and subsequent substitution of one or two copies.

#. Sites were found by those primers reported by Wallner et al. (2013).
