# Supplementary material for: Ancient Patrilineal Lines and Relatively High ECAY Diversity Preserved in Indigenous Horses Revealed With Novel Y-Chromosome Markers
Source: Front Genet. 2020 May 21;11:467. doi: 10.3389/fgene.2020.00467 (PMC7253630; doi:10.3389/fgene.2020.00467)
Supplement: FIGURE S1 — Geographic distributions of sampled Chinese indigenous horse populations. [file Data_Sheet_1.ZIP › supplementary materials/Table S3. Primers for real-time PCR.doc]

**Table S3. Primers for real-time PCR**

| Locus | Size (bp) | Annealing temperature (℃) | primers（5’-3’） |
| --- | --- | --- | --- |
| 165 | 109 | 60 | F: CCTGAGTAATGAAGCAGACAATAG  R: GAGACAGATGAGACTCCAGAAATG |
| 17 | 230 | 60 | F: GCACTAGTGCAGAAGGTGGCAGTGTAG  R: GCAGTGCTGTTATGCGCAGACCAGTGT |
| 102 | 151 | 58 | F: GCAAGGTCCTGCCTGGGATGAA  R: TCTAGATGGTAGCTTACGGCTTC |
| 103 | 142 | 58 | F: GCCCTGTGGTGGCATTTCA  R: TGACTCCATCCAACGGAGGAGCC |
| 9 | 111 | 60 | F: GTTACAAGGACTCCCAGG  R: GGATAGCATCTTCACCAC |
| 14 | 91 | 56 | F: CTAAGGGAGATATTCTGCACG  R: GAATGACCGATGCCTGTGTTG |
| 1 | 169 | 60 | F: AGCGGGGAAAGACATTGACT  R: ATTCGCAGCGTTGACCACAG |
| 12 | 159 | 60 | F: CAAGATTACCTCCTGGGACAAC  R: AATCACTGTTATTCACGCCTGC |
| 132 | 136 | 60 | F: TGATTCAATAAACCCAAACCCC  R: GGCTTACTGACCATCTCTTCTG |
| 133 | 175 | 60 | F: TGGCAGAGACAATACGGGAGAT  R: GGCACCCAGGATGTGTGTTCTC |
| 4 | 170 | 60 | F: GGAGGTAGAGTATCCCCATCG  R: TTCCCCACTGTGTATGTCTGT |
| GAPDH | 137 | — | F: CCAGAACATCATCCCTGCTT  R: AAGCAGGATCAGCACCACTT |
