# Supplementary material for: Ancient Patrilineal Lines and Relatively High ECAY Diversity Preserved in Indigenous Horses Revealed With Novel Y-Chromosome Markers
Source: Front Genet. 2020 May 21;11:467. doi: 10.3389/fgene.2020.00467 (PMC7253630; doi:10.3389/fgene.2020.00467)
Supplement: FIGURE S1 — Geographic distributions of sampled Chinese indigenous horse populations. [file Data_Sheet_1.ZIP › supplementary materials/Table S4. The information of primers for pyrosequencing.doc]

**Table S4.** The information of primers for pyrosequencing

| Locus | Primer *(5’- 3’) | Size (bp) | Annealing temperature (°C) |
| --- | --- | --- | --- |
| 165 | F: GCCCTTGCTGAAGTGGACATTATC  R: TCCGGGCAGTGCTGTTATG  S: GCAGCTGGAGACAAA | 135 | 51 |
| 17 | F: TCTCACCACATTTGTGTCAGAGTC  R: GCTTTGTCTTTTGGGCTTTTTATG  S: TTCAAACTGTGCCCA | 182 | 52 |
| 102 | F: TGTCCTCAGACCGCTTGG  R: ACTTCCTTAGGGGGCAGGC  S: AGGTGGCGAGGCTGG | 85 | 61 |
| 103 | F: AGTACAGGGCCCACGTAGTCCA  R: GGGAAAAGAAGGAGCCACTCG  S: AGTCCACGTCCCTAAC | 88 | 50 |
| 9 | F: CCCACTGATATCCAACTTTAACG  R: TTGGCATGTTTAGGAGAATCTG  S: CATCTTCACCACTCTGTATA | 166 | 50 |
| 1 | F: TGGCACAGTAAAATAGTCTCCATG  R: GCCCTCAATAGTCTGTGTTGAA  S: TTGAAACTGACTTCAGC | 69 | 49 |

* F, forward primer; R: reverse primer; S, primer used for determining the allele-specific copy number variations at the polymorphic sites.
